# Activated phosphoinositide 3-kinase δ syndrome caused by PIK3CD mutations: expanding the phenotype

**DOI:** 10.1186/s12969-024-00955-7

**Published:** 2024-01-29

**Authors:** Peiwei Zhao, Juan Huang, Huicong Fu, Jiali Xu, Tianhong Li, Xiankai Zhang, Qingjie Meng, Lei Zhang, Li Tan, Wen Zhang, Hebin Chen, Xiaoxia Lu, Yan Ding, Xuelian He

**Affiliations:** 1 Precision Medical Center, Tongji Medical College, Wuhan Children’s Hospital (Wuhan Maternal and Child Healthcare Hospital, Huazhong University of Science & Technology, Wuhan, 430016 China; 2 Department of Pathology, Tongji Medical College, Wuhan Children’s Hospital (Wuhan Maternal and Child Healthcare Hospital, Huazhong University of Science & Technology, Wuhan, 430016 China; 3 Department of Respiratory Medicine, Tongji Medical College, Wuhan Children’s Hospital (Wuhan Maternal and Child Healthcare Hospital, Huazhong University of Science & Technology, Wuhan, 430016 China; 4 Department of Clinical Laboratory, Tongji Medical College, Wuhan Children’s Hospital (Wuhan Maternal and Child Healthcare Hospital, Huazhong University of Science & Technology, Wuhan, 430016 China; 5 Department of Rheumatology and Immunology, Tongji Medical College, Wuhan Children’s Hospital (Wuhan Maternal and Child Healthcare Hospital, Huazhong University of Science & Technology, Wuhan, 430016 China

**Keywords:** Activated phosphoinositide 3-kinase δ syndrome, *PIK3CD*, Immunodeficiency, Phenotype, ANCA-associated vasculitis

## Abstract

**Background:**

Germline heterozygous gain-of-function (GOF) mutations in the *PIK3CD* gene lead to a rare primary immunodeficiency disease known as activated phosphoinositide 3-kinase (PI3K) δ syndrome type 1(APDS1). Affected patients present a spectrum of clinical manifestations, particularly recurrent respiratory infections and lymphoproliferation, increased levels of serum immunoglobulin (Ig) M, Epstein-Barr virus (EBV) and cytomegalovirus (CMV) viremia. Due to highly heterogeneous phenotypes of APDS1, it is very likely that suspected cases may be misdiagnosed.

**Methods:**

Herein we reported three patients with different clinical presentations but harboring pathogenic variants in *PIK3CD* gene detected by trio whole-exome sequencing (trio-WES) and confirmed by subsequent Sanger sequencing.

**Results:**

Two heterozygous mutations (c.3061G > A, p.E1021K and c.1574 A > G, p.E525G) in *PIK3CD* (NM_005026.3) were identified by whole exome sequencing (WES) in the three patients. One of two patients with the mutation (c.3061G > A) presented with abdominal pain and diarrhea as the first symptoms, which was due to intussusception caused by multiple polyps of colon. The patient with mutation (c.1574 A > G) had an anti-neutrophil cytoplasmic antibody (ANCA)-associated vasculitis (AAV)-like clinical manifestations, including multisystemic inflammation, acute nephritic syndrome, and positive perinuclear ANCA (p-ANCA), thus the diagnosis of ANCA-AAV was considered.

**Conclusions:**

Our study expands the spectrums of clinical phenotype and genotype of APDS, and demonstrates that WES has a high molecular diagnostic yield for patients with immunodeficiency related symptoms, such as respiratory infections, multiple ecchymosis, ANCA-associated vasculitis, multiple ileocecal polyps, hepatosplenomegaly, and lymphoid hyperplasia.

**Trial registration:**

Retrospectively registered.

## Introduction

Activated phosphoinositide 3-kinase (PI3K) δ syndrome (APDS) is an autosomal dominant primary immunodeficiency(PID) caused by gain-of-function (GOF) variants in *PIK3CD* (NM_005026.3) and *PIK3R1* [1]. Variants in *PIK3CD* gene coding PI3K δ catalytic subunit p110δ lead to APDS1, while variants in *PIK3R1* gene coding regulatory subunit p85α are associated with APDS2 [2]. The phenotypes of APDS are highly heterogeneous, ranging from asymptomatic or subclinical infection, to serious immunodeficiency. The majority of patients present with recurrent sinopulmonary infections, elevated levels of serum immunoglobulin (Ig) M, low levels of serum IgG, lymphoproliferation, autoinflammatory disease, Epstein-Barr virus (EBV) and cytomegalovirus (CMV) viremia, as well as Epstein-Barr virus (EBV) and non-EBV driven malignancies [3–5].

PIK3CD, which belongs to the class IA PI3Ks, is mainly expressed in lymphocytes and plays a crucial role in immune cell function [6, 7]. p110δ encoded by *PIK3CD* gene contains multiple functional domains(adaptor-binding domain, ras-binding domain, C2, Helical, N-lobe and C-lobe) [8]. To date there are 12 pathogenic germline GOF variants identified in *PIK3CD* gene, including E81K, G124D, N344K, R405C, C416R, Y524N, E525G, E525A, E525K, R929C, E1021K and E1025G, of which E1021K is the most frequently reported mutation in patients with APDS1 [9–13]. In addition, bi-allelic loss-of-function (LOF) mutations in *PIK3CD* leads to immunodeficiency 14B (OMIM #619,281) [14], Therefore, too little or too much PI3Kδ activity would lead to immunodeficiency.

Herein, we report three unrelated Chinese patients with heterogeneous manifestations: one patient with hepatosplenomegaly, short stature and recurrent infetation, one patient presented with multiple ecchymosis, with a medical history of abdominal pain and diarrhea caused by intussusception, and one patient with recurrent respiratory infections, nephritic syndrome, and ANCA-associated vasculitis. All these patients carry one heterozygous mutations (c.3061G > A, p.E1021K or c.1574 A > G, p.E525G) in *PIK3CD* gene identified by whole exome sequencing (WES).

## Materials and methods

### Study subjects

Three male patients suspected of immunodeficiency, aged from 5 years to 7 years, were recruited in this study. Upon obtaining informed consent, clinical data of patients were collected and peripheral bloods were withdrawn from the patients and their parents. Genomic DNA was extracted from peripheral blood of the patients using the MicroElute Genomic DNA Kit (OMEGA) according to the manufacturer’s instructions. Respiratory and intestinal mucosas were obtained from the patients for pathological examination. This study has been approved by the institutional review board of Wuhan Children’s Hospital, Tongji Medical College, Huazhong University of Science & Technology (NO.2021R060-E01).

### Whole exome sequencing

Whole exome sequencing (WES) and subsequent data analysis were performed with the help of a third party medical laboratory (Chigene Lab, Beijing China). Databases from the 1000 genomes project, dbSNP, and ExAC were applied for investigating minor allele frequency. The variants were annotated using the OMIM, HGMD, and ClinVar databases for pathogenicity analysis. *PIK3CD* gene variants were confirmed by Sanger sequencing using site specific primers in patients and their parents. The pathogenicity of variants was interpreted according to American College of Medical Genetics and Genomics (ACMG) guidelines, and all *PIK3CD* variants in this study were described using the reference sequence (NM_005026).

### Immunohistochemistry

Bronchial mucosa or intestinal mucosa tissue from the patients with APDS were processed individually by preparing 4 μm sections of the formalin-fixed and paraffin-embedded tissues. Standard immunohistochemistry was performed. The following primary antibodies were used for staining the tissue sections: anti-CD3 antibody (1:100), anti-CD20 antibody (1:100), anti-AKT antibody (CST, 1:100). The retrieval of antigen in the tissue sections was performed in Tris-buffer (pH9.0) using autoclave. Antigen visualization was done with peroxidase-diaminobenzidine reactions.

## Results

### Case presentation

Patient 1, a 4-year and 10-month old Chinese boy, was admitted to our hospital for hepatosplenomegaly for two years, short stature for one and half year, and cough and fever for more than one week. He is the third child from healthy nonconsanguineous parents without related familial history of genetic diseases, and no obvious abnormality was found at birth. This patient had repeated respiratory tract infections during the last 3 years, with a frequency of 1–2 times a month.

At presentation, he was febrile (38.9^o^C), anemic appearance, short stature (102 cm, less than 3rd ), low weight (15 kg, less than3rd ). In addition, enlarged lymph nodes could be palpated under the jaw, neck and behind the ear. Blood results revealed a slightly elevated level of white blood cell (WBC) count (10.12 x 10^9^/L), a significantly decreased hemoglobin (84 g/L) and serum IgG level (0.08 g/L), as well as abnormal lymphocyte proportion in peripheral blood detected by flow cytometry (shown in Table 1). EBV and CMV DNA in peripheral blood were negative. Bone marrow biopsy showed granulocytic hyperplasia and small cell low pigment changes in mature erythrocytes. Chest computer tomography (CT) revealed bilateral pneumonia and partial consolidation. Abdominal CT showed an increase in the volume of liver and spleen, and multiple enlarged lymph nodes in the mesentery and retroperitoneum, with the diameters of about 4–15 mm, and no obvious abnormal density was found in the parenchyma.

**Table 1:** Clinical characteristics of patients with APDS1 caused by *PIK3CD* gene

Clinical manifestation	P1	P2	P3	Reference value
Age	5Y	7Y10M	7Y2M	
Gender	M	M	M	
Main manifestations	fever, hepatosplenomegaly	abdominal pain intussusception	pneumonia, hematuria	
Recurrent infection	yes	no	yes	
Recurrent fever	yes	no	yes	
Hepatosplenomegaly	yes	no	no	
Lymphadenopathy	yes	yes	yes	
Other exceptions	swelling of both knees	multiple polyps of intestine, thrombocytopenia	otitis media, allergic rhinitis	
WBC(10^9/L)	10.12	11.18	9.4	5.5–12
Hgb (g/L)	84	122	113	110–149
PLT(10^9/L)	146	20	131	100–378
LYM(10^9/L)	2.05	1.42	1.27	1.7–4.7
LYM(%)	29.0	16.6	13.5	22–57
CD16 + CD56 (n/ul)	64	192	833	210–1514
CD19+ (n/ul)	55	169	194	240–1317
CD3 + T (n/ul)	1740	721	1307	805–4459
CD3 + CD8 + T (n/ul)	1048	254	803	314–2080
CD3 + CD4 + T (n/ul)	450	379	470	345–2350
IgG (g/L)	0.08	11	8.21	3.48–7.01
IgM (g/L)	1.6	3.57	1.53	0.42–1.73
IgA (g/L)	0.26	2.01	0.45	0.28–1.73
Hematuria	(-)	(-)	(++)	(-)
Proteinuria	(-)	(-)	(+)	(-)
p-ANCA	/	/	(+)	(-)
c-ANCA	/	/	(-)	(-)
MPO (AU/ml)	/	/	(-)	< 16
Proteinase 3 (AU/ml)	/	/	(-)	< 16
EBEA-IgG	(-)	(-)	(+)	(-)
EBV-IgG	(-)	(+)	(+)	(-)
CMV-IgM	(-)	(+)	(-)	(-)
CMV-IgG	(-)	(+)	(+)	(-)

After admission, a laparotomy revealed enlarged liver and spleen, as well as small intestinal mesenteric lymph nodes. Two pieces of tissues (0.8 x 0.6 x 0.5 cm, respectively) from the margins of liver and spleen, and three larger lymph nodes, were taken for pathological examinations. During exploratory laparotomy, a Meckel diverticulum was observed and removed from about 50 cm away from the ileocecal region, opposite to the mesentery of the small intestine, after obtaining the consent of his parents. The biopsy of the surgical specimen demonstrated that the mucosal lymphoid tissue proliferation and positive EBER (EBV-encoded RNAs) in situ hybridization in a few cells, indicating lymphatic hyperplasia possibly caused by EBV infection, with no evidence of malignancy.

One month after discharge, the patient presented with swelling of both knee joints, especially the left knee, affecting normal activities. Magnetic Resonance Imaging (MRI) showed abnormal signal of fluid accumulation focusing in the bilateral knee joint cavities and the left knee suprapatellar capsule, as well as slightly swelling muscles surrounding and thickening and strengthening of the synovial membrane in the left knee joint. The swelling relieved after taking naproxen 10 ~ 15 mg/(kg.d) orally twice for three days.

**Fig. 1:**
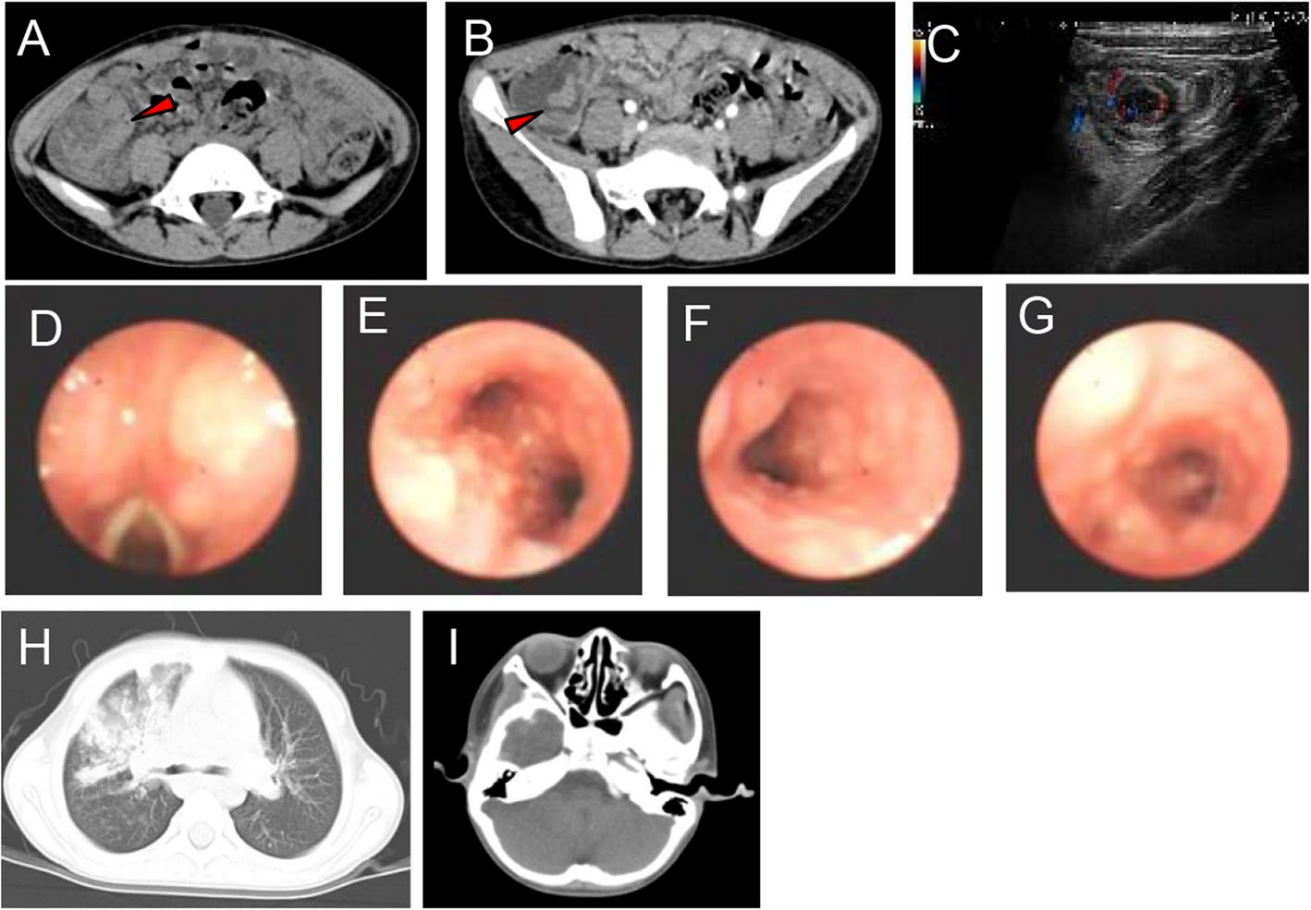
Clinical phenotype of patients with APDS1.  Ileocecal intussusception and polypoid soft tissue nodule in ileocecal intestinal cavity revealed by MRI (**A** and **B**) and target ring sign detected by ultrasound (**C**) in patient 2. Mucosal nodule lymphoid hyperplasia from pharynx to the entire airway revealed by bronchoscopy in patient 3 (**D**-**G**); Pulmonary parenchymal lesions and patchy infiltration, and maxillary sinusitis in patient 3 by CT scan (**H**, **I**)

Patient 2, an 8.5-year old boy, presented our allergy clinic with multiple ecchymosis for half day. The patient had no abnormality at birth and was in normal physical condition. At the age of 2 months old, he had been diagnosed as thrombocytopenia. At 5 years, he underwent cervical lymphadenectomy, and lymph node biopsy demonstrated reactive hyperplasia. At 7 years old, he received colonoscopy and polypectomy due to abdominal pain and diarrhea due to intussusception caused by multiple ileocecal polyps (Fig. 1A, B and C).

At presentation, he had multiple ecchymosis on both lower and limbs, scattered petechiae on limbs and trunk, and enlarged superficial lymph nodes on both side of the neck, with the largest one of 2cm x 2cm. His height and weight were normal. Physical examination and imaging identified no convincing enlargement of liver and spleen. Blood tests showed a low blood platelet count 72 (10^9/L), a normal count of red blood cells 4.53(10^12/L) and a slightly elevated count of WBC (5.55 x 10^9^/L), the levels of serum IgA, IgG and IgM were slightly increased (gamma globulin infusion one week before this test), and abnormal lymphocyte proportion in peripheral blood detected by flow cytometry (Table 1). In addition, the serum detection of CMV IgM was positive, however, the concentrations of CMV and EBV DNA were less than 4.00E + 02 copies/ml and 2.00E + 03 copies/ml, respectively, excluding the infection of CMV and EBV. Although the patient was initially diagnosed with immune thrombocytopenia, his medical history was pointing to immunodeficiency disease, thus, peripheral blood was drawn from the patient and his parents for trios WES.

Patient 3, a 7-year and 2-month old boy, was referred to the pneumology department due to recurrent respiratory infections. He was the only child born to a nonconsanguineous parents. His mother had recurrent infection and allergic rhinitis during her childhood. The patient had a history of multiple recurrent infections that had started from one month after birth, including bronchopneumonia, otitis media, amygdalitis, conjunctivitis, EBV infection, skin infections (mural abscess and furunculosis) and recurrent purpura, and even septicopymeia. At the age of 6 years old, he had recurrent hematuria, and the renal function tests showed elevated serum creatinine and uric acid, proteinuria and erythrocyturia, and the diagnosis of acute nephritic syndrome was considered. His parents refused to have renal puncture. The other clinical laboratory examinations were shown in Table 1. An ultrasound scan of kidneys and bilateral ureters was negative. The lung CT scan revealed right lung pneumonia with alveolar consolidation of right upper lobe (Fig. 1H). Bronchoscope examination found bronchial mucosal hypertrophy with follicular hyperplasia, and stenosis of the basal segment bronchial opening in the lower lobe of the right lung (Fig. 1D and G). Pathological analysis of tissues taken from the bronchus showed partial tissues were significantly compressed and a large number of inflammatory cells, mainly lymphocytes and plasma cells, in the epithelium and stroma. The immunohistochemistry staining revealed positive for CD3 and CD20, scattered positive CD138 cells. In addition, autoimmune serology was positive for perinuclear ANCA (p-ANCA) (the titer was 128) and negative for cytoplasmic ANCA (c-ANCA) and proteinase 3, according o the clinical manifestations and positive p-ANCA, the diagnosis of ANCA-associated vasculitis (AAV) was considered.

### Genetic analysis

**Fig. 2:**
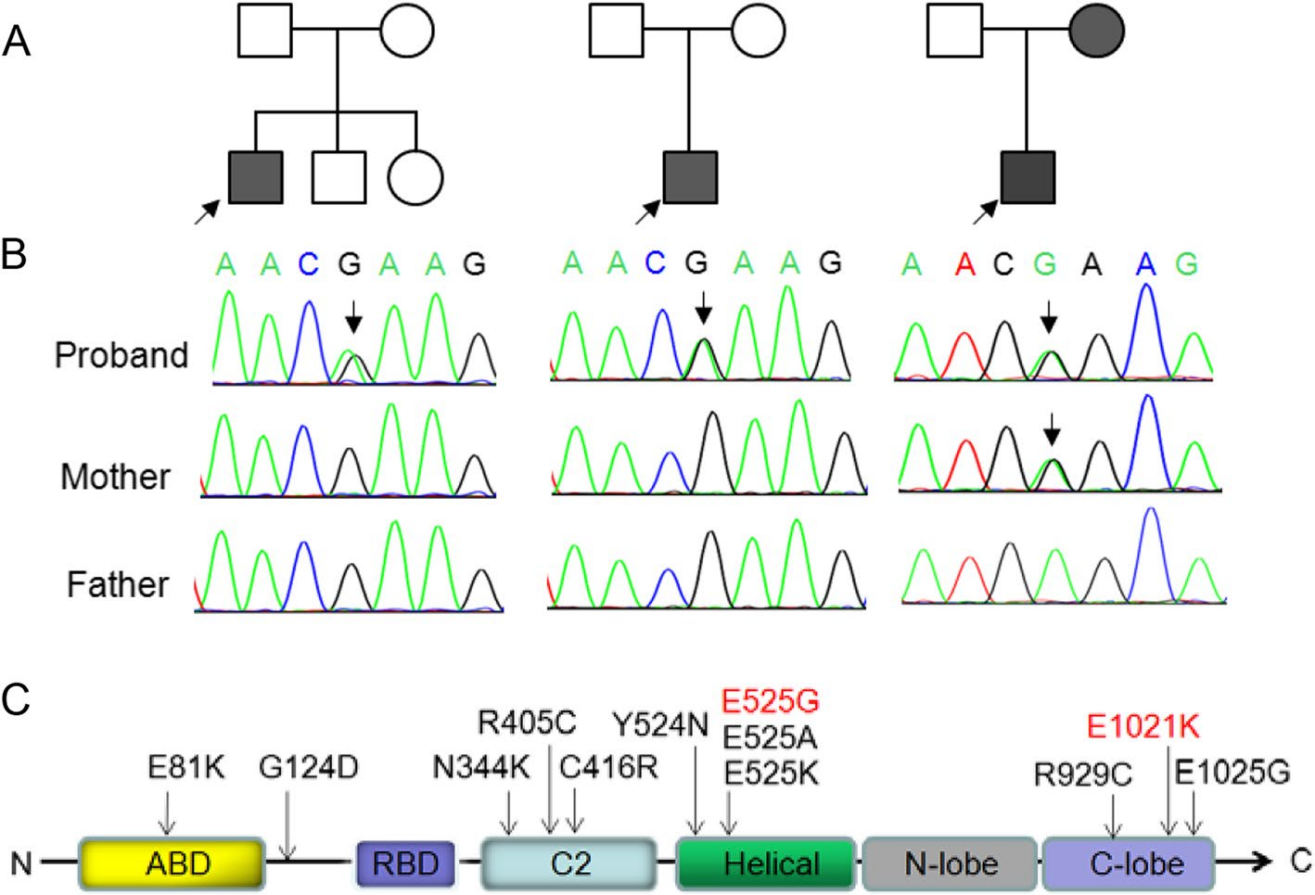
Genealogical tree of three families (**A**). Sanger sequencing of *PIK3CD* gene variants in three patients of this study (**B**). Scheme of the distribution of *PIK3CD* gain of function mutations, and the mutations labeled in red were reported in our patient (C)

In order to determine the cause of the patient’s recurrent sinopulmonary infections, hepatosplenomegaly, and/or mucosal follicular hyperplasia for a clear diagnosis, trios-WES was conducted. Bioinformatic analysis was performed to identify candidate variants according to filtering strategies based on population frequency, variant classification, and variant functional damaging prediction, including Polymorphism Phenotyping v2, Sorting Intolerant From Tolerant, and Combined Annotation Dependent Depletion. A heterozygous mutation (c.3061G > A, p.E1021K) was found in *PIK3CD* gene (NM_005026) in patient 1 and patient 2. Sanger sequencing showed that this mutation was *de novo* and their parents did not carry this mutation. Another heterozygous mutation (c.1574 A > G, p.E525G) was found in patient 3, and this mutation was inherited from his mother (Fig. 2A and B). These two variants are pathogenic according to ACMG guidelines for variant classification (PS1 + PM1 + PM2 + PM5 + PP3). The diagnosis of APDS was made based on the patients’ clinical presentations and genotypes.

### Immunohistochemistry

**Fig. 3:**
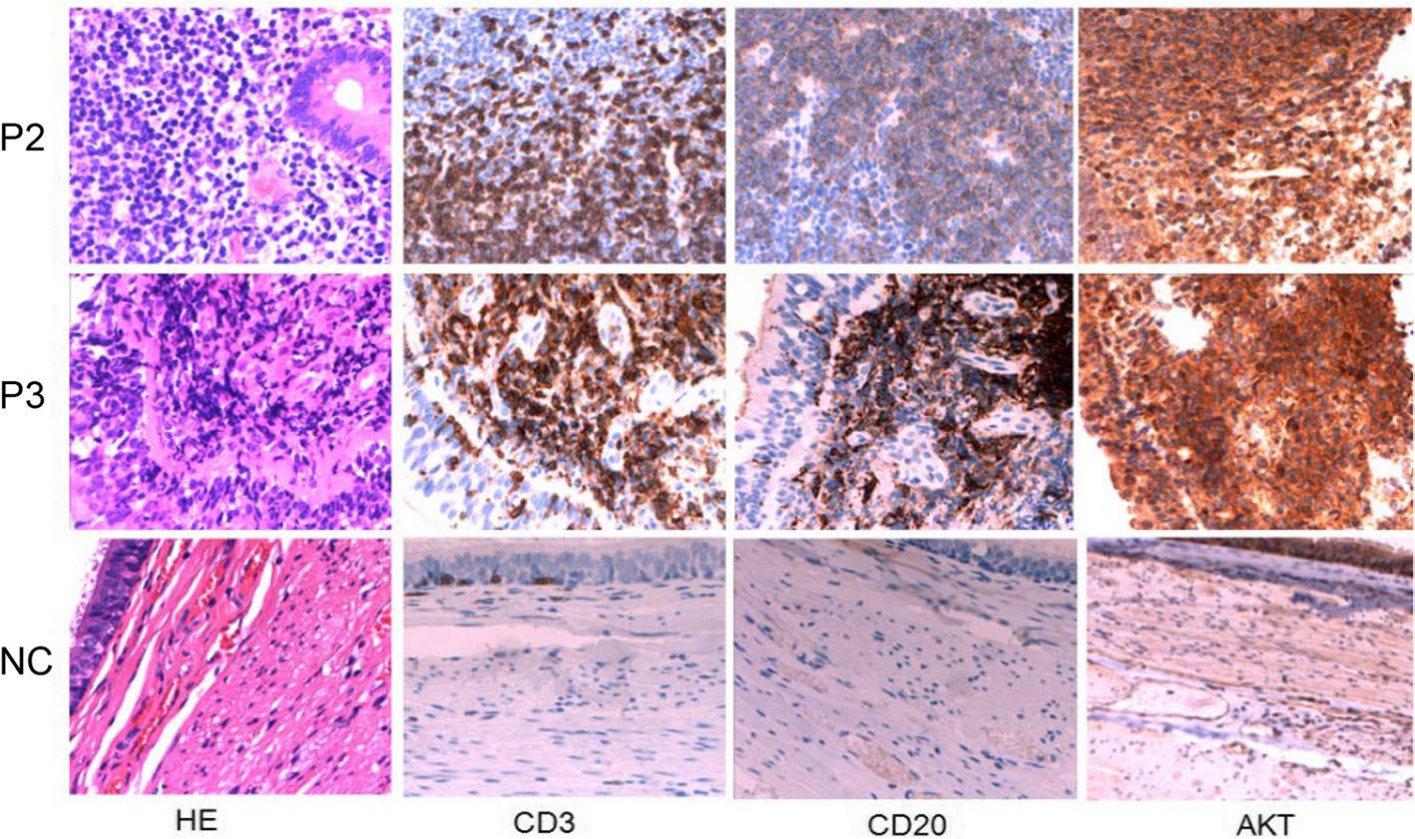
The numbers of CD3 + T cells and CD20 + B cells and the expression level of AKT protein increased significantly. (NC: normal control; P: patient)

Compared with the control sample, the number of lymphocytes in the mucosa of patients was significantly increased. The polyp-like protrusion of intestinal mucosa in patient 2 was taken. Biopsy showed obvious hyperplasia of mucosal lymphoid tissue, formation of lymphoid follicles and proliferative lesions caused by chronic stimulation of the intestine. A large number of inflammatory cells mainly composed of lymphocytes and plasma cells could be seen in the bronchial mucosa epithelium and stroma of patient 3, which suggests chronic inflammatory changes. CD3 and CD20 are expressed in the cell membrane of lymphocytes and where CD3 labeled T lymphocytes while CD20 labeled B lymphocytes (Fig. 3). Immunohistochemical results showed that the number of T and B lymphocytes in mucosal tissue was significantly increased compared with control group. In addition, we found that the expression level of AKT in patients’ tissues was significantly increased which prompted for activation of AKT signal pathway in tissues (Fig. 3).

## Discussion

GOF mutations in the p110δ subunit of *PIK3CD* cause an immunodeficiency characterized by recurrent infections leading to structural lung abnormalities including bronchiectasis, lymphoproliferation, and EBV or CMV viremia [15]. Almost all patients with APDS have benign lymphoid tissue hyperplasia (e.g. hepatosplenomegaly and lymph node enlargement). Lymph node enlargement, persistent or recurrent, often occurs within 1 year of age, usually limited to the infection site. Approximately 34% of APDS patients have autoimmune complications, including hemocytopenia glomerulonephritis, juvenile idiopathic arthritis, thyroiditis and sclerosing cholangitis. In this work, we reported that three patients presented with recurrent infections, hepatosplenomegaly, or abdominal pain, and had novel or inherited germline mutations in *PIK3CD* gene. Based on clinical manifestations and the genetic findings, a diagnosis of APDS was considered.

Activation of PI3Kδ results in phosphorylation of phosphatidylinositol 3,4-bisphosphate (PIP2) to yield phosphatidylinositol 3,4,5-trisphosphate (PIP3), and the latter actives AKT and other related pathways to induce proliferation and inhibit apoptosis [16]. In this study, we investigated the expression level of AKT in the mucosa of patients was significantly increased, indicating the possible activation of AKT signaling pathway. However, due to the sample processing method, phosphorylated AKT could not be detected.

AAV affects systemic small vessels and is accompanied by the existence of ANCAs in the serum of patients [17]. ANCAs play important roles in the pathogenesis of AAV because they induce excessive activation of neutrophils and damage to small blood vessels. AAV includes granulomatosis with polyangiitis (GPA), MPA, eosinophilicgranulomatosis with polyangiitis and drug-induced type [17]. Lu et al. reported two pediatric APDS patients with recurrent lung infections, sinusitis, hematuria, renal involvement and positive c-ANCA, manifesting classic symptoms of GPA [2]. Currently, our patient 3 with p.E525G mutation had no typical changes of GPA, no nasal polyp and obstructive airway disease, eosinophils less than 1 x 10^9^/L, and positive c-ANCA. However, the hypertrophy of bronchial mucosa and stenosis of the basal segment bronchial opening were observed by bronchoscope examination. At present, it is difficult to determine whether the diagnosis is MPA or GPA. In addition, the phenotypes of patients are variable, even carrying the same mutation in the same family, as his mother only showed recurrent infection and allergic rhinitis at childhood. All these cases indicate that autoimmune conditions, including AAV, are common manifestations of APDS. Therefore, genetic testing should be performed on these patients with AAV to exclude the possibility of primary immunodeficiency.

Literature review revealed that clinical phenotypes of gastrointestinal diseases were relatively rare in patients with APDS. Previously only two adult patients with *PIK3CD* mutations had primary sclerosing cholangitis [5]. Patient 2 in this study, presented with abdominal pain and diarrhea due to intussusception, and was diagnosed as APDS by genetic testing. This case was considered as secondary intussusception caused by a large number of polyps in the ileocecal region.

In summary, the clinical phenotypes of APDS diseases are highly heterogeneous. We reported three cases of APDS with rare clinical phenotypes, including joint swelling, ANCA associated AAV, multiple ecchymosis and secondary intussusceptions. Our cases further support the fact that autoimmune conditions are important manifestations of APDS and emphasize the importance of molecular detection in assisting the diagnosis of rare diseases.

## Data Availability

The data underlying this article will be shared on reasonable request to the corresponding author.

## Abbreviations

GOF: Germline heterozygous gain-of-function
APDS1: Activated phosphoinositide 3-kinase (PI3K) δ syndrome type 1
EBV: Epstein-Barr virus
CMV: Cytomegalovirus
WES: Whole-exome sequencing
ANCA: Anti-neutrophil cytoplasmic antibody (ANCA)-associated vasculitis
AAV: ANCA-associated vasculitis
p-ANCA: Perinuclear ANCA
MPA: Microscopic polyangiitis
PID: Primary immunodeficiency
ACMG: American College of Medical Genetics and Genomics
WBC: White blood cell
